# Duloxetine-Induced Antidiuresis in Rats with Lithium-Induced Nephrogenic Diabetes Insipidus

**DOI:** 10.3390/life14081012

**Published:** 2024-08-15

**Authors:** Sua Kim, Chor Ho Jo, Gheun-Ho Kim

**Affiliations:** 1 Biomedical Research Institute, Hanyang University College of Medicine, 222-1 Wansimni-ro, Seongdong-gu, Seoul 04763, Republic of Korea; tndkfl@hanyang.ac.kr (S.K.); screen41@hanmail.net (C.H.J.); 2 Department of Internal Medicine, Hanyang University College of Medicine, 222-1 Wansimni-ro, Seongdong-gu, Seoul 04763, Republic of Korea

**Keywords:** antidepressant, aquaporin-2, cAMP, hyponatremia, tolvaptan, vasopressin-2 receptor

## Abstract

Antidepressants, including duloxetine, are a significant cause of drug-induced hyponatremia, which can disrupt the continuation of medication. Tolvaptan is beneficial for correcting hyponatremia caused by the syndrome of inappropriate antidiuresis, but its impact on duloxetine-induced hyponatremia remains unknown. We used male Sprague-Dawley rats to examine the impact of duloxetine treatment on lithium-induced nephrogenic diabetes insipidus (Li-NDI) and to evaluate whether the results were reversed by co-treatment with tolvaptan. To induce Li-NDI, lithium chloride (40 mmol lithium/kg dry food) was administered for 2 weeks. Duloxetine (50 mg/kg/day) and tolvaptan (10 mg/kg/day) were also administered in food to assess their individual effects over the same period. At the end of each animal experiment, kidneys were harvested to measure levels of cAMP, vasopressin-2 receptor (V2R), cAMP-responsive element binding protein 1 (CREB-1), aquaporin-2 (AQP2), and prostaglandin E2 (PGE2). Water diuresis was induced in the Li-NDI rats, and duloxetine treatment reduced polyuria while increasing urine osmolality. Duloxetine treatment prevented the decrease in total AQP2, AQP2 phosphorylation at serine 256, and CREB-1 phosphorylation in Li-NDI rats. The V2R mRNA level was also reduced in Li-NDI rats and restored by duloxetine treatment. In the subsequent experiment, the decreased water diuresis in Li-NDI rats treated with duloxetine was reversed by co-treatment with tolvaptan. Tolvaptan co-treatment also reversed the changes in AQP2 protein and CREB-1 phosphorylation in the renal cortex and medulla. The decreased cAMP levels in Li-NDI rat kidneys were elevated by duloxetine treatment, and this elevation was reversed by co-treatment with tolvaptan. However, the elevated PGE2 levels in Li-NDI rat kidneys were not affected by either duloxetine alone or tolvaptan co-treatment. In conclusion, antidiuresis was induced by duloxetine in Li-NDI and reversed by tolvaptan co-treatment through alterations in the V2R-cAMP-AQP2 pathway. These findings could underlie the mechanism of duloxetine-induced hyponatremia and suggest the potential usefulness of tolvaptan in treating drug-induced hyponatremia.

## 1. Introduction

Antidepressants, such as duloxetine, are a major cause of drug-induced hyponatremia. Selective serotonin reuptake inhibitors (SSRIs) and selective serotonin–norepinephrine reuptake inhibitors (SSNRIs) are typical types of currently used antidepressants, and hyponatremia can occur in patients using either SSRIs or SSNRIs [1]. The mechanism by which SSRIs, such as fluoxetine [2] and sertraline [3], can induce hyponatremia has been explored in experimental studies, and it was found that they upregulate aquaporin-2 (AQP2) in the kidney without arginine vasopressin. However, how SSNRIs, such as duloxetine, cause water retention through the kidney has yet to be investigated. The incidence of hyponatremia was reported to be more common with SSNRIs than with SSRIs [1].

Duloxetine is currently prescribed to treat depression and anxiety disorders. Although it is clinically beneficial, its use is limited by side effects, including hyponatremia [1,4]. Duloxetine-induced hyponatremia has been considered to occur from the syndrome of inappropriate antidiuretic hormone secretion (SIADH) [5,6,7,8], but it is unclear whether duloxetine stimulates arginine vasopressin release from the posterior pituitary gland. Renal water retention can occur without the stimulation of arginine vasopressin, and spontaneous or drug-induced activation of the vasopressin-2 receptor (V2R) is known to cause nephrogenic syndrome of inappropriate antidiuresis (NSIAD) [9].

To resolve drug-induced hyponatremia, all potentially associated agents should be discontinued. However, in some situations, the offending agent cannot be discontinued because no alternative treatment is available [10]. Given that several drugs inducing hyponatremia act as V2R agonists [11], we hypothesized that the antidiuretic effect in drug-induced hyponatremia could be reversed by selective V2R antagonists like tolvaptan. We tested this possibility by administering duloxetine with and without tolvaptan to rats with lithium-induced nephrogenic diabetes insipidus (Li-NDI). We conducted two separate animal experiments to determine whether and how duloxetine treatment causes antidiuresis in Li-NDI and to evaluate whether the antidiuresis and its associated molecular mechanisms were reversed by tolvaptan co-treatment.

## 2. Materials and Methods

### 2.1. Animal Experiments

Male pathogen-free Sprague-Dawley rats weighing 180–200 g (Orient Bio Inc., Seongnam, Republic of Korea) were used in two animal experiments. In each experiment, all rats were fed a slurry mixture of regular rat chow (18 g/200 g BW/d) and deionized water (18 mL/200 g BW/d), and they were housed in metabolic cages with freely accessible drinking water. In animal experiment 1, the rats were randomly divided into four groups: normal controls (n = 6), Li-NDI rats (n = 6), duloxetine-treated normal rats (n = 6), and duloxetine-treated Li-NDI rats (n = 6). To induce Li-NDI, lithium chloride (40 mmol lithium/kg dry food) was administered for 2 weeks. Duloxetine (50 mg/kg/day; CAS No. 136434-34-9, Sigma-Aldrich, St. Louis, MO, USA) was orally administered in food to the treated groups during the same period. This dose was based on the previous paper, which described the renal effects of duloxetine in female Sprague-Dawley rats at oral doses of 20 and 60 mg/kg [12].

In animal experiment 2, the V2R antagonist tolvaptan was used to determine if the effects of duloxetine treatment in Li-NDI rats could be reversed by blocking V2R. The rats were randomly divided into the following four groups: controls (n = 6), Li-NDI rats (n = 6), duloxetine-treated Li-NDI rats (n = 6), and duloxetine/tolvaptan-cotreated Li-NDI rats (n = 6). Administration of lithium and duloxetine was conducted in the same manner as in animal experiment 1. Additionally, tolvaptan was orally administered in food (10 mg/kg BW/d) during the same period. This dose was based on the previous publications regarding the use of tolvaptan in acute and chronic rat models of hyponatremia [13,14,15,16].

At the end of each animal experiment, plasma and 24 h urine samples were collected for chemical analysis (AU680, Beckman Coulter, Brea, CA, USA). For the collection of blood samples, the rats were anesthetized by intramuscular injection of tiletamine/zolazepam (Zoletil, 30 mg/kg BW) and xylazine (10 mg/kg BW). These experimental protocols were approved by the Institutional Animal Care and Use Committee of Hanyang University (HY-IACUC-23-0004; HY-IACUC-23-0005).

### 2.2. Immunoblot Analysis

Manually dissected slices of the kidney were homogenized in a buffer containing 250 mM sucrose, 10 mM triethanolamine, 1 µg/mL leupeptin, and 0.1 mg/mL phenylmethylsulfonyl fluoride, titrated to pH 7.6. Protein samples were separated by sodium dodecyl sulphate–polyacrylamide gel electrophoresis, and the proteins were electrophoretically transferred from unstained gels to nitrocellulose membranes (Bio-Rad, Hercules, CA, USA). After being blocked with 5% skim milk in PBS-T (80 mM Na_2_HPO_4_, 20 mM NaH_2_PO_4_, 100 mM NaCl, 0.1% Tween-20, pH 7.5) for 1 h, the membranes were probed overnight at 4 °C with one of the following primary antibodies: rabbit polyclonal anti-AQP2 (#AQP-002, Alomone Labs, Jerusalem, Israel), rabbit polyclonal anti-pSer256-AQP2 (#ab111346, Abcam, Cambridge, MA, USA), rabbit polyclonal anti-CREB-1 (#06-863, Sigma-Aldrich), mouse monoclonal anti-p-CREB-1 (#sc-81486, Santa Cruz Biotechnology, Dallas, TX, USA), and mouse monoclonal anti-β-actin (#A5441, Sigma-Aldrich). The secondary antibodies used were goat anti-rabbit (#111-035-003, Jackson ImmunoResearch, West Grove, PA, USA) and donkey anti-mouse (#715-035-151, Jackson ImmunoResearch) IgG conjugated to horseradish peroxidase. The sites of antibody–antigen reactions were visualized using luminol-based enhanced chemiluminescence (WEST-ZOL^®^Plus, Intron Biotechnology, Sungnam, Republic of Korea). The band densities of the immunoblots were quantified with densitometry using a laser scanner and Quantity One software (version 5.2, Bio-Rad Laboratory, Berkeley, CA, USA).

### 2.3. Immunohistochemistry

The kidneys were perfused with 1% phosphate-buffered saline (PBS) by retrograde perfusion via the abdominal aorta to remove blood, followed by perfusion with periodate-lysine-paraformaldehyde (PLP; 0.01 M NaIO_4_, 0.075 M lysine, 2% paraformaldehyde, in 0.0375 M Na_2_HPO_4_ buffer, pH 6.2) for 10 min for kidney fixation. After the completion of perfusion, each kidney was sliced into 4 mm thick pieces and immersed in a 2% PLP solution overnight at 4 °C. Renal tissues were fixed in 10% neutral-buffered formalin and embedded in paraffin. Embedded kidney slices were sectioned to a thickness of 4 µm using a microtome. Sections were deparaffinized using a graded series of ethanol and then treated with 3% H_2_O_2_ for 30 min to eliminate endogenous peroxidase activity. Heat-induced epitope retrieval was performed using a citrate buffer (pH 6.0). Sections were then blocked with 10% normal donkey serum for 1 h and incubated overnight at 4 °C with primary antibodies. The next day, the sections were washed in PBS and incubated for 2 h using a DAKO Envision kit (Dako, Glostrup, Denmark) at room temperature. The samples were washed with Tris buffer and then incubated in a solution containing 0.05% 3,3′-diaminobenzidine and 0.033% H_2_O_2_. Tissues were counterstained with hematoxylin, and slides were mounted with Canadian balsam (Sigma-Aldrich).

### 2.4. Quantitative Polymerase Chain Reaction (qPCR)

Total RNA was isolated from whole rat kidneys using TRIzol^®^ reagent (Life Technologies, Carlsbad, CA, USA) and quantified by spectrophotometry. A cDNA synthesis was performed using 3 μg of RNA and SuperScript^®^ III reverse transcriptase (Life Technologies). For qPCR, 100 ng of cDNA served as the template for PCR amplification using Brilliant SYBR Green QPCR Master Mix according to the manufacturer’s instructions (FastStart DNA Master SYBR Green I; Roche Molecular Biochemicals, Mannheim, Germany). Serial dilutions (1 ng/μL to 1 fg/μL) of cDNA were used as the template to generate a standard curve. Nested primers were used to amplify the standard and kidney cDNA samples (Table 1). The standard and unknown samples were amplified in duplicate in 96-well plates. The thermal profile of the LightCycler^®^ instrument (Roche Molecular Biochemicals) was optimized by initiating denaturation for 10 min at 95 °C, followed by 45 amplification cycles of 10 s at 95 °C, 10 s at 60 °C, and 10 s at 72 °C. The comparative Ct method was used to determine the relative amounts of target mRNA, expressed for each sample as a percentage of the glyceraldehyde 3-phosphate dehydrogenase mRNA level. The Ct ratios were analyzed using LightCycler^®^ software (version 4.05). Specificity was verified through a post-run melting curve analysis.

### 2.5. Cyclic Adenosine Monophosphate (cAMP) and Prostaglandin E2 (PGE2) Measurement from Rat Kidneys

Intracellular levels of cAMP and PGE2 were measured in renal medullae prepared from animal experiment 2. For the cAMP measurement, fresh medullary homogenates were centrifuged at 1500× *g* for 10 min. The supernatant was transferred to a clean test tube, and the cAMP level was determined according to the assay protocol using a Cyclic AMP Select ELISA kit (#501040, Cayman Chemical, Ann Arbor, MI, USA). The protein content of each sample was measured, and the results are expressed as picomoles per milligram of protein. For the PGE2 measurement, fresh medullary homogenates were centrifuged at 8000× *g* for 10 min. The supernatant was transferred to a clean test tube, and the PGE2 level was determined following the assay protocol using a Prostaglandin E2 Express ELISA kit (#501040, Cayman Chemical). The protein content of each sample was measured, and the results are expressed as picograms per milligram of protein.

### 2.6. Statistics

The data are presented as mean ± standard deviation (SD). Kruskal–Wallis nonparametric testing was used to assess the significance of differences among groups, and post-hoc comparisons between two groups were performed using the Mann–Whitney *U* test (GraphPad Prism 10, La Jolla, CA, USA). To facilitate immunoblot and qPCR comparisons, protein band density or relative mRNA values were normalized by dividing them by the mean value of the control group; *p* values less than 0.05 were considered statistically significant.

## 3. Results

### 3.1. Animal Experiment 1: Effects of Duloxetine Treatment in Li-NDI

Table 2 summarizes the physiological data obtained at the end of animal experiment 1. As expected, urine output was significantly increased after 2 weeks of lithium ingestion. This polyuria was associated with a significant decrease in urine osmolality, indicating water diuresis. However, plasma sodium and osmolality were not significantly affected by lithium-induced polyuria. Duloxetine treatment in Li-NDI reduced urine output from 56.5 ± 7.8 to 28.7 ± 6.4 mL/day/100 g BW (*p* = 0.004). Urine osmolality increased from 227 ± 38 to 336 ± 95 mOsm/kg/H_2_O (*p* = 0.036). Interestingly, duloxetine treatment alone (without lithium) reduced urine output (3.8 ± 0.4 versus 4.9 ± 0.8 mL/d/100 g BW, *p* = 0.025) and increased urine osmolality (2181 ± 148 versus 1918 ± 170 mOsm/kg/H_2_O, *p* = 0.021) compared with controls. 

**Figure 1:**
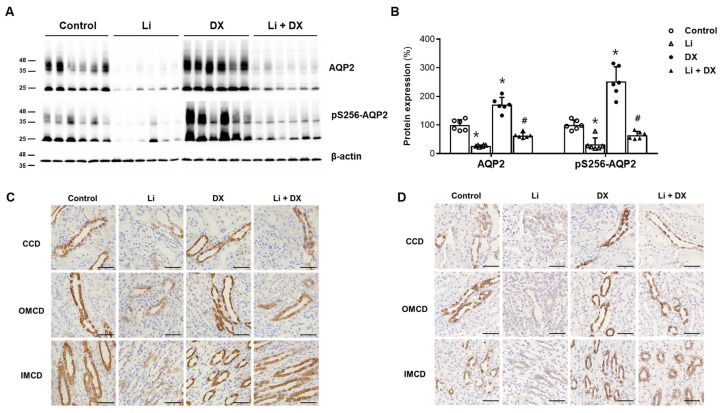
Effects of duloxetine treatment on aquaporin-2 (AQP2) protein expression in lithium-induced nephrogenic diabetes insipidus. Immunoblots of AQP2 and pS256-AQP2 are shown; each lane was loaded with a protein sample from a different rat kidney (**A**). Densitometric analysis revealed that the protein levels of AQP2 and pS256-AQP2 were significantly reduced by lithium treatment (Li) compared to controls, but were restored by lithium/duloxetine co-treatment (Li + DX). The levels of AQP2 and pS256-AQP2 proteins increased in normal rats treated with duloxetine (DX) compared to the control group (**B**). Immunohistochemistry is shown for AQP2 (**C**) and pS256-AQP2 (**D**) in rat kidneys. Along the cortical collecting duct (CCD), outer medullary collecting duct (OMCD), and inner medullary collecting duct (IMCD), differences in AQP2 labeling among groups were consistent with those in the immunoblot analyses. Each bar in the densitometry results represents the mean ± standard deviation (**B**). *, *p* < 0.05 vs. control; ^#^, *p* < 0.05 vs. Li by the post hoc Mann–Whitney *U* test. Scale bars, 50 μm.

The expression of total AQP2 protein and AQP2 protein phosphorylated at serine 256 (pS256-AQP2) was compared among the animal groups (Figure 1). Immunoblot analysis revealed a significant decrease in total AQP2 and pS256-AQP2 in the Li-NDI rats compared to the controls. However, the total AQP2 (from 27 ± 3 to 63 ± 8%, *p* = 0.001) and pS256-AQP2 (from 32 ± 21 to 64 ± 12%, *p* = 0.021) in the Li-NDI rats increased with duloxetine treatment. Consistent with urinary responses, treatment with duloxetine alone (without lithium) led to an upregulation of total AQP2 (171 ± 23%, *p* = 0.001) and pS256-AQP2 (252 ± 47%, *p* = 0.001) compared with controls.

These immunoblot findings were confirmed by immunohistochemistry. Figure 1C illustrates that AQP2 labelling was remarkably attenuated in Li-NDI rats compared with controls, and duloxetine treatment reinstated AQP2 labelling along the collecting duct. Figure 1D shows distinct apical pS256-AQP2 labelling, especially in rats treated with duloxetine alone (without lithium). The faint pS256-AQP2 immunostaining along the collecting duct in Li-NDI rats was also restored by duloxetine treatment.

**Figure 2:**
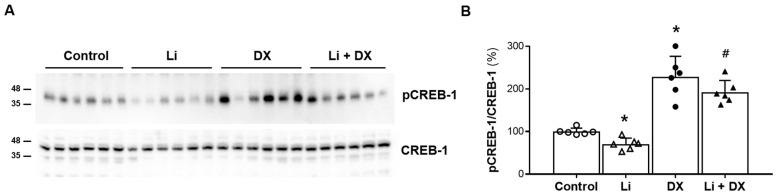
Effects of duloxetine treatment on cAMP response element binding protein-1 (CREB-1) expression in lithium-induced nephrogenic diabetes insipidus. Immunoblots of CREB-1 and phosphorylated CREB-1 (pCREB-1) are shown, with each lane loaded with a protein sample from a different rat kidney (**A**). Densitometric analysis indicates that the protein level of pCREB-1 was reduced by lithium treatment (Li) compared to controls, but was restored by lithium/duloxetine co-treatment (Li + DX). The pCREB-1 level increased in duloxetine-treated normal rats (DX) compared with controls (**B**). Each bar in the densitometry results represents the mean ± standard deviation. *, *p* < 0.05 vs. control; ^#^, *p* < 0.05 vs. Li by the post hoc Mann–Whitney U test.

Similar responses were noted in the immunoblot analysis for CREB-1 (Figure 2). CREB-1 phosphorylation was reduced in Li-NDI rats (70 ± 13%, *p* = 0.002) compared with controls, and duloxetine treatment restored CREB-1 phosphorylation (192 ± 25%, *p* = 0.001). Consistent with the upregulation of AQP2, treatment with duloxetine alone (without lithium) increased CREB-1 phosphorylation (228 ± 44%, *p* = 0.001) compared with controls.

**Figure 3:**
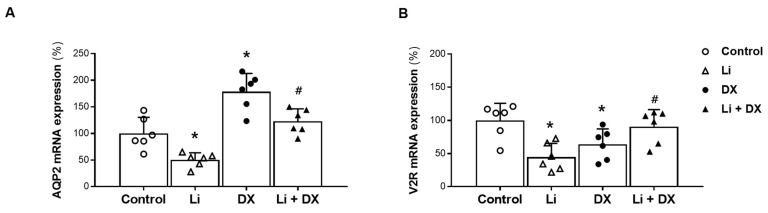
Effects of duloxetine treatment on mRNA levels of aquaporin-2 (AQP2) and vasopressin-2 receptor (V2R) in lithium-induced nephrogenic diabetes insipidus. Quantitative PCR analyses of AQP2 (**A**) and V2R (**B**) were performed using whole kidneys. Compared with controls, the mRNA levels of AQP2 and V2R were decreased by lithium treatment (Li) and increased by lithium/duloxetine co-treatment (Li + DX). Each bar represents the mean ± standard deviation. *, *p* < 0.05 vs. control; ^#^, *p* < 0.05 vs. Li by the post hoc Mann–Whitney *U* test.

Figure 3 shows the mRNA levels of AQP2 and V2R in the qPCR results. Both AQP2 and V2R mRNA decreased in Li-NDI rats compared with the controls, and duloxetine treatment restored them. AQP2 levels increased from 50 ± 13 to 128 ± 23% (*p* = 0.001), and V2R levels increased from 45 ± 21 to 91 ± 25% (*p* = 0.013). AQP2 mRNA was upregulated by duloxetine treatment alone, but V2R mRNA was not.

### 3.2. Animal Experiment 2: Reversal of Duloxetine Effects by Tolvaptan Co-Treatment in Li-NDI

Animal experiment 2 was performed to confirm the results of animal experiment 1 and to determine whether the effects of duloxetine treatment in Li-NDI were mediated by V2R and reversible with tolvaptan co-treatment. Changes in urine output and urine osmolality in response to lithium with and without duloxetine were very similar to those in animal experiment 1 (Table 3). The reduced polyuria in duloxetine-treated Li-NDI rats (17.5 ± 5.5 mL/day/100 g BW) was reversed by tolvaptan co-treatment (64.7 ± 5.3 mL/day/100 g BW, *p* = 0.004). Consistent with this, co-treatment with tolvaptan reduced urine osmolality in Li-NDI rats treated with duloxetine (from 542 ± 247 to 179 ± 19 mOsm/kg/H_2_O, *p* = 0.004). 

**Figure 4:**
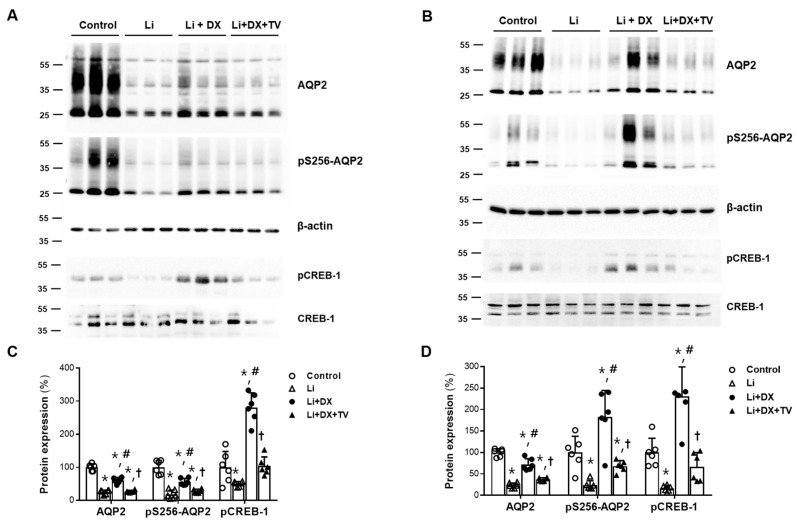
Reversal of duloxetine effects by tolvaptan co-treatment in lithium-induced nephrogenic diabetes insipidus. Immunoblots of AQP2, pS256-AQP2, CREB-1, and pCREB-1 are shown from the renal cortex (**A**) and medulla (**B**); each lane was loaded with a protein sample from a different rat kidney. Densitometric analysis revealed that the protein levels of AQP2, pS256-AQP2, and pCREB-1 were significantly reduced by lithium treatment (Li) compared to controls, but were restored by lithium/duloxetine co-treatment (Li + DX). However, all changes in both the cortex (**C**) and medulla (**D**) were reversed by the addition of tolvaptan (Li + DX + TV). Each group contained six rats, and the densitometry results are presented as the mean ± standard deviation. *, *p* < 0.05 vs. control; ^#^, *p* < 0.05 vs. Li; ^†^, *p* < 0.05 vs. Li + DX by the post hoc Mann–Whitney *U* test.

In animal experiment 2, immunoblot analyses for AQP2 and CREB-1 were performed after dividing the kidneys into cortices and medullae (Figure 4). In both the cortex and medulla, the reduced protein expression of total AQP2 and pS256-AQP2 in Li-NDI rats was significantly increased by duloxetine treatment. The recovery of total AQP2 and pS256-AQP2 was completely blocked by tolvaptan co-treatment (Figure 4A,B). The decreased phosphorylation of CREB-1 in Li-NDI rats was also elevated by duloxetine treatment in both the cortex and medulla. Consistent with AQP2 changes, the upregulation of CREB-1 phosphorylation in both the cortex and medulla was completely blocked by co-treatment with tolvaptan (Figure 4C,D).

**Figure 5:**
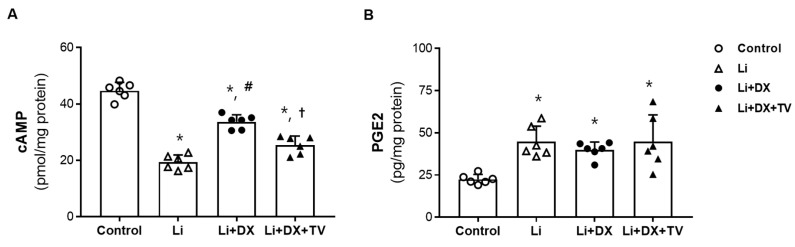
Effects of duloxetine treatment on renal cAMP and prostaglandin E2 (PGE2) in lithium-induced nephrogenic diabetes insipidus. The cAMP (**A**) and PGE2 (**B**) contents were determined using enzyme immunoassays from renal medullae. Compared with controls, cAMP levels decreased with lithium treatment (Li), improved with lithium/duloxetine co-treatment (Li + DX), and decreased again with the addition of tolvaptan (Li + DX + TV). On the other hand, PGE2 levels increased with lithium treatment (Li) and remained unchanged with the addition of duloxetine or duloxetine/tolvaptan co-treatment. Each bar represents the mean ± standard deviation. *, *p* < 0.05 vs. control; ^#^, *p* < 0.05 vs. Li; ^†^, *p* < 0.05 vs. Li + DX by the post hoc Mann–Whitney *U* test.

Finally, we compared the cAMP and PGE2 contents in the renal medulla among the animal groups. Compared with controls, Li-NDI rats had a lower level of cAMP (44.7 ± 3 versus 19.3 ± 2.6 pmol/mg protein, *p* = 0.001), which was increased by duloxetine treatment (33.7 ± 3 pmol/mg protein, *p* = 0.001) and reversed by tolvaptan co-treatment (25.4 ± 3 pmol/mg protein, *p* = 0.001). Thus, the changes in cAMP levels in response to lithium, lithium + duloxetine treatment, and lithium + duloxetine/tolvaptan co-treatment were very similar to the changes in AQP2 and CREB-1 protein levels (Figure 5A). On the other hand, the responses of PGE2 were not concordant. Compared with controls, Li-NDI rats had a higher level of PGE2 (22.5 ± 3 versus 44.8 ± 9 pg/mg protein, *p* = 0.001), and this PGE2 level was not affected by duloxetine treatment or duloxetine/tolvaptan co-treatment (Figure 5B).

## 4. Discussion

In this study, we report two novel findings about the induction and correction of renal water retention. First, a representative SSNRI (duloxetine) caused water retention by upregulating V2R-cAMP-AQP2 signaling in the kidney. Secondly, the antidiuretic effect induced by duloxetine in Li-NDI was reversed by co-administering tolvaptan. Each finding has significant clinical implications for the pathophysiology and treatment of duloxetine-induced hyponatremia. We did not measure serum potassium and urine chloride in our rats because Li-NDI is a water balance disorder. However, we cannot exclude the possibility of potential disturbances in potassium or acid-base balances.

Duloxetine exerted a potent antidiuretic effect in our animal model of Li-NDI. It effectively reduced the urine volume by half and increased the urine osmolality by 1.5 times. Consistent with this, the downregulation of AQP2 in Li-NDI rats was significantly improved by duloxetine administration. The V2R mRNA, which was suppressed in Li-NDI rats, was also restored by duloxetine. This suggests that V2R might play a pivotal role in duloxetine-induced antidiuresis. We did not directly test the protein expression of V2R because valid antibodies to V2R were unavailable.

Interestingly, duloxetine alone also increased urine concentration in normal rats, which could be linked to duloxetine-induced hyponatremia in humans. We also found that duloxetine increased total AQP2 protein and mRNA levels, as well as pS256-AQP2 and CREB-1 phosphorylation, in rat kidneys. Thus, duloxetine administration stimulated both short-term (phosphorylation) and long-term (transcription) regulation of AQP2.

In another animal experiment, we further investigated the role of V2R in duloxetine-induced antidiuresis by using tolvaptan. Tolvaptan selectively blocks V2R and effectively induces diuresis in patients with the syndrome of inappropriate antidiuresis [17]. However, its efficacy in drug-induced hyponatremia has not been explored.

We found that the effects of duloxetine-induced antidiuresis in Li-NDI were nullified by tolvaptan co-treatment, which restored both urine output and urine osmolality to the levels of Li-NDI. Consistent with this, the upregulation of AQP2 and phosphorylation of CREB-1 induced by duloxetine were also inhibited by co-treatment with tolvaptan. The responses were very similar in both the renal cortex and medulla. Thus, we conclude that V2R mediates duloxetine-induced antidiuresis in Li-NDI. In addition, we suggest considering the feasibility of using tolvaptan to treat drug-induced hyponatremia.

Although a number of drugs, including sildenafil and statins, have been shown to cause antidiuresis in animal models of Li-NDI [11], they seldom induce hyponatremia in clinical practice. Notably, drugs that can induce hyponatremia in humans have a common mechanism of action: AQP2 upregulation via V2R stimulation [9]. Chlorpropamide is the standard for drug-induced hyponatremia. It was previously shown to competitively bind to V2R within the rat renal tubular basolateral membrane [18] and increase V2R density in rat renal papillary membranes [19]. We previously demonstrated that haloperidol, sertraline, carbamazepine, and cyclophosphamide directly affect V2R in the collecting duct and increase AQP2 levels by activating the cAMP–protein kinase A pathway [3,20]. Our current results add duloxetine to the list of V2R agonists.

**Figure 6:**
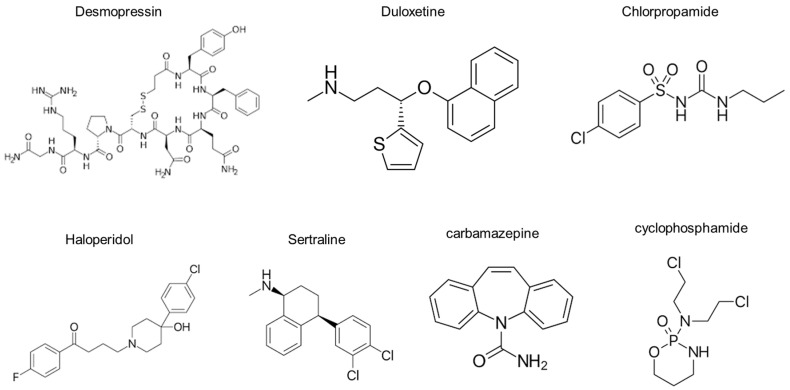
The chemical structures of agents known to induce renal water retention.

Desmopressin is the prototype of the V2R agonist, and its chemical structure is very similar to that of arginine vasopressin. It is a synthetic, deaminated analogue of arginine vasopressin, bearing a substitution in position 8, where d-Arg replaces l-Arg [21]. Compared with desmopressin, duloxetine has a simpler structure. Additionally, duloxetine does not appear to have any characteristic chemical structures in common with chlorpropamide, haloperidol, sertraline, carbamazepine, and cyclophosphamide (Figure 6). Further studies are required to explore how these agents interact with the V2R.

We tested whether PGE2 is involved in the pathways affected by duloxetine because PGE2 regulates water reabsorption in the collecting duct through AQP2 trafficking [22,23]. Consistent with a previous study [24], our Li-NDI rat kidneys exhibited a lower level of cAMP and a higher level of PGE2 compared to the controls. The lithium-induced decrease in cAMP production has also been reported in humans. In healthy volunteers, the ability to concentrate urine was reduced after four weeks of lithium therapy, along with decreased excretion of urinary AQP2 and cAMP [25]. In this study, duloxetine treatment of Li-NDI rats enhanced urine concentration in association with increased renal cAMP production and AQP2 expression. We confirmed that duloxetine participates in the V2R-cAMP-AQP2 pathway because the increased renal cAMP production was reversed by co-treatment with tolvaptan.

On the other hand, the lithium-induced increase in PGE2 synthesis was not altered by either duloxetine treatment or duloxetine/tolvaptan co-treatment. Thus, the pathway for duloxetine action in the collecting duct does not involve PGE2-AQP2 signaling. This finding appears to be consistent with the study by Fenton and colleagues, which demonstrated V2R-independent upregulation of AQP2 through E-prostanoid receptor agonism [26].

Based on our results in this study, we propose tolvaptan as an adjunctive therapy for drug-induced hyponatremia. When psychotropic drugs must be continued despite hyponatremia, concurrent use of tolvaptan might be useful. However, this new therapeutic option for drug-induced hyponatremia needs to be tested in clinical trials.

## 5. Conclusions

We demonstrated that duloxetine induced antidiuresis in Li-NDI and that this effect was reversed by co-treatment with tolvaptan, which altered the V2R-cAMP-AQP2 pathway. These findings may explain the mechanism behind duloxetine-induced hyponatremia and indicate the potential benefit of using tolvaptan to treat drug-induced hyponatremia.

## Figures and Tables

**Table 1:** Primer sequences for qPCR.

Gene	Forward (F) and Reverse (R) Primer Sequences	PCR Product (bp)	GenBank Accession No.
AQP2	F 5′-CATGTCTCCTTCCTTCGAGCTG-3′ R 5′-CCCCACGGATTTCTACTGGAGT-3′	125	NM_012909.2
V2R	F 5′-GCTCTTCATCTTTGCTCAGCGT-3′ R 5′-TCCAGGTGACATAGGCACGAA-3′	110	NM_019136

qPCR, quantitative polymerase chain reaction; AQP2, aquaporin-2 water channel; V2R, vasopressin-2 receptor.

**Table 2:** Functional data from animal experiment 1.

Parameter	Control (n = 6)	Li (n = 6)	DX (n = 6)	Li + DX (n = 6)
Body weight (g)	316 ± 13	261 ± 6 *	299 ± 7 *	266 ± 13
Urine output (mL/day/100 g BW)	4.9 ± 0.8	56.5 ± 7.8 *	3.8 ± 0.4 *	28.7 ± 6.4 ^#^
Urine osmolality (mOsm/kg H_2_O)	1918 ± 170	227 ± 38 *	2181 ± 148 *	336 ± 95 ^#^
Urine sodium (mmol/day/100 g BW)	0.89 ± 0.14	1.37 ± 0.20 *	0.84 ± 0.12	0.90 ± 0.20 ^#^
Urine creatinine (mg/day/100 g BW)	3.38 ± 0.80	4.64 ± 0.90 *	3.13 ± 0.36	3.03 ± 0.63 ^#^
Plasma osmolality (mOsm/kg H_2_O)	307 ± 10	314 ± 9	308 ± 6	308 ± 3
Plasma sodium (mmol/L)	141 ± 3	143 ± 2	143 ± 3	144 ± 2
Plasma creatinine (mg/dL)	0.31 ± 0.03	0.35 ± 0.05	0.34 ± 0.05	0.33 ± 0.03

Data are mean ± SD. *, *p* < 0.05 vs. control; ^#^, *p* < 0.05 vs. Li by the post hoc Mann-Whitney *U* test.

**Table 3:** Functional data from animal experiment 2.

Parameter	Control (n = 6)	Li (n = 6)	Li + DX (n = 6)	Li + DX + TV (n = 6)
Body weight (g)	344 ± 13	288 ± 20 *	300 ± 10	283 ± 3 ^†^
Urine output (mL/day/100 g BW)	6.7 ± 0.8	67.6 ± 5.7 *	17.5 ± 5.5 ^#^	64.7 ± 5.3 ^†^
Urine osmolality (mOsm/kg H_2_O)	1610 ± 165	188 ± 21 *	542 ± 247 ^#^	179 ± 19 ^†^
Urine sodium (mmol/day/100 g BW)	0.78 ± 0.06	1.14 ± 0.13 *	0.57 ± 0.09 ^#^	0.93 ± 0.08 ^†^
Urine creatinine (mg/day/100 g BW)	3.31 ± 0.30	4.07 ± 0.46 *	2.62 ± 0.46 ^#^	3.28 ± 0.35 ^†^
Plasma osmolality (mOsm/kg H_2_O)	321 ± 7	315 ± 6	321 ± 9	317 ± 5
Plasma sodium (mmol/L)	144 ± 2	142 ± 1	142 ± 7	143 ± 2
Plasma creatinine (mg/dL)	0.39 ± 0.07	0.50 ± 0.09	0.51 ± 0.13	0.45 ± 0.03

Data are mean ± SD. *, *p* < 0.05 vs. control; ^#^, *p* < 0.05 vs. Li; ^†^, *p* < 0.05 vs. Li + DX by the post hoc Mann–Whitney *U* test.

## Data Availability

The raw data supporting the conclusion of this article will be made available by the authors, without undue reservation.
